# Neuroprotective Effect of Physiological Circulating Phenolic Metabolites Against Oxidative Stress- and Neuroinflammation-Induced in Human Cellular Models

**DOI:** 10.3390/ijms27146487

**Published:** 2026-07-21

**Authors:** Beatriz Garay-Mayol, Silvia Navarro-Orcajada, Sabrina Poveda-Lora, Maria Alexandra Brito, Juan Antonio Giménez-Bastida, María Ángeles Ávila-Gálvez, Antonio González-Sarrías

**Affiliations:** 1 Research Group on Quality, Safety and Bioactivity of Plant Foods, CEBAS-CSIC, Campus de Espinardo, 30100 Murcia, Spain; bgaray@cebas.csic.es (B.G.-M.); snavarro@cebas.csic.es (S.N.-O.); spoveda@cebas.csic.es (S.P.-L.); jgbastida@cebas.csic.es (J.A.G.-B.); 2 Research Institute for Medicines (iMed.ULisboa), Faculty of Pharmacy, Universidade de Lisboa, Av. Prof. Gama Pinto, 1649-003 Lisbon, Portugal; abrito@ff.ulisboa.pt; 3 Department of Pharmaceutical Sciences and Medicines, Faculty of Pharmacy, Universidade de Lisboa, Av. Prof. Gama Pinto, 1649-003 Lisbon, Portugal

**Keywords:** phenolic compounds, blood–brain barrier, microglia cells, neuroblastoma cells, HBMECs, ROS levels

## Abstract

Growing evidence indicates that circulating phenolic metabolites can reach brain tissue and exert neuroprotective effects; however, their combined activity at physiological concentrations remains poorly understood. This study evaluated three metabolite mixtures designed according to previously reported plasma and brain metabolite profiles following a Mediterranean (poly)phenol-rich intervention (Mix Eq, Mix %C, and Mix %T), together with their individual constituent metabolites, in human cellular models of oxidative stress, neuroinflammation, and blood–brain barrier (BBB) dysfunction. All physiological mixtures significantly protected SH-SY5Y neuroblastoma cells against H_2_O_2_-induced oxidative stress by improving cell viability and reducing intracellular ROS production in a concentration-dependent manner, with cotreatment showing greater efficacy than pretreatment. Individual metabolites also showed protective effects, although none matched the overall efficacy of the mixtures. In HMC3 cells, the mixtures moderately attenuated LPS- and TNF-α-induced inflammatory responses, reducing IL-6 and IL-8. Finally, in HBMECs, TNF-α increased BBB permeability and cytokine release, whereas Mix %C significantly attenuated TNF-α-induced sodium fluorescein permeability. However, none of the mixtures significantly reduced IL-6 or IL-8 secretion in this BBB model. Overall, physiologically relevant combinations of circulating phenolic metabolites showed greater neuroprotective activity than individual metabolites, supporting the contribution of additive or cooperative interactions to the benefits associated with Mediterranean diet-derived phenolics.

## 1. Introduction

The progressive ageing of the global population has positioned neurodegenerative diseases (NDs) among the most important public health and socioeconomic problems in developed countries. Currently, more than 55 million people worldwide are living with dementia or Alzheimer’s disease, and 12 million individuals have Parkinson’s disease [1]. Furthermore, these numbers are projected to rise a dramatic increase from 25.5 million cases in 2000 to nearly 114 million by 2050 [2,3]. NDs are characterized by the progressive loss of neurons and function, leading to impaired motor and cognitive functions, including dementia and behavioural alterations [4,5]. Although the underlying mechanisms vary among disorders, several common pathological features have been identified, including oxidative stress, chronic neuroinflammation and blood–brain barrier (BBB) dysfunction [6]. Among these, oxidative stress is recognized as a key contributor to neurodegeneration. Excessive production of reactive oxygen species (ROS), including hydrogen peroxide (H_2_O_2_), promotes mitochondrial dysfunction, oxidative damage, and neuronal death, thereby contributing to cognitive decline [7,8]. Similarly, neuroinflammation plays a central role in the pathogenesis of ND. This process is primarily mediated by the activation of microglial cells, which respond to intrinsic and extrinsic stimuli, including infections, protein aggregates, and cellular damage signals. Activated microglia release pro-inflammatory cytokines such as interleukin (IL)-6 and IL-8, as well as additional inflammatory mediators that exacerbate neuronal injury and oxidative damage [9,10]. In parallel, BBB disruption is also a key pathological feature of neurodegeneration. BBB leakage facilitates the infiltration of peripheral immune cells, inflammatory mediators, and neurotoxic molecules into the brain parenchyma, amplifying neuroinflammatory and oxidative processes and contributing to the loss of neuronal homeostasis [11,12].

Despite significant advances in research, effective therapeutic interventions capable of preventing or slowing the progression of ND remain unavailable, and the potential for early diagnosis is still limited. Furthermore, evidence indicates that pathological brain changes associated with ND may begin up to two decades before the onset of clinical symptoms [13,14]. Consequently, prevention has emerged as one of the most promising approaches to reduce the burden of these disorders, highlighting the importance of identifying modifiable lifestyle factors that contribute to the maintenance of brain health. Substantial epidemiological and experimental studies evidence suggests that adherence to plant-based dietary patterns, especially the Mediterranean diet, which is characterized by a high consumption of fruits, vegetables, legumes, and whole grains, is associated with a lower risk of cognitive decline and ND [15,16,17].

Furthermore, many of these studies have attributed the observed neuroprotective effects to the presence of diverse bioactive compounds in plant-derived foods, particularly dietary (poly)phenols, which may exert their beneficial effects through multiple mechanisms, including antioxidant activity via free radical scavenging and reduction in ROS, as well as anti-inflammatory actions through the modulation of pro-inflammatory markers [16,18]. However, despite the promising therapeutic potential of dietary (poly)phenols as a non-pharmacological strategy against ND, important limitations remain, as many studies continue to overlook key aspects such as bioavailability, metabolism, and the capacity of these compounds to reach target tissues [19]. Moreover, several challenges continue to hinder our understanding of the neuroprotective potential of these metabolites. First, few studies have identified metabolized metabolites in both perfused and non-perfused brain tissue using physiologically relevant animal models while avoiding non-dietary doses, direct intravenous administration of metabolites, or the use of nano-formulations [20,21,22,23,24], and second, although recent studies have reported neuroprotective effects for several metabolites at physiologically relevant concentrations, most investigations have focused on individual compounds [25,26,27,28,29,30]. These approaches do not adequately reproduce the complex metabolic exposure that occurs following dietary intake, where multiple circulating metabolites coexist and may interact through additive or synergistic mechanisms.

In a recent study by our group [31] provided novel insights into the metabolic fate and brain distribution of phenolic compounds following the administration of a Mediterranean diet-derived (poly)phenol-rich extract mixture containing pomegranate, lemon, orange, grape, and olive components. Using pharmacokinetic analyses in Sprague-Dawley rats, 39 circulating phenolic metabolites were detected in plasma, of which 19 were also detected in perfused brain tissue, including hydroxytyrosol and tyrosol sulfates, ellagic acid, resveratrol sulfate, dihydrocaffeic acid, and several derived metabolites. Importantly, in vitro BBB transport experiments suggested enhanced transport efficiency when these compounds were evaluated as metabolite mixtures rather than as individual molecules.

Therefore, the present study aimed to investigate the neuroprotective potential of physiologically relevant phenolic metabolite mixtures, at physiological concentrations, designed according to previously reported plasma and brain metabolite profiles following consumption of a Mediterranean (poly)phenol-rich intervention [31]. Specifically, the neuroprotective effect of three mixtures differing in composition and relative abundance (Mix Eq, Mix %C, and Mix %T) were evaluated in human neuronal models of oxidative stress (H_2_O_2_-induced neurotoxicity in neuroblastoma SH-SY5Y cells), neuroinflammation (LPS/TNF-α-stimulated microglial HMC3 cells), and TNF-α-induced BBB damage. In addition, the effects of the 14 individual metabolites identified in these mixtures were also assessed in order to determine the potential contribution of specific metabolites to the biological activities observed for the combined formulations.

## 2. Results

### 2.1. Physiological Mixtures and Individual Circulating Phenolic Metabolites Protect SH-SY5Y Cells from H_2_O_2_-Induced Cytotoxicity and Reactive Oxygen Species (ROS) Production

**Figure 1:**
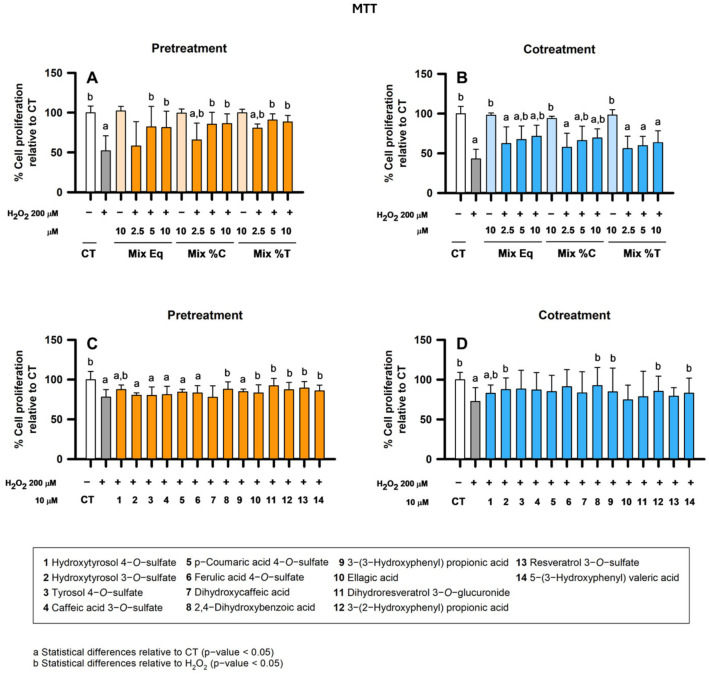
Effects of physiological phenolic metabolite mixtures and individual phenolic metabolites on cell proliferation in SH-SY5Y cells. The cells were exposed to H_2_O_2_ (200 µM) either following (**A**,**B**) physiological mixtures under pretreatment and cotreatment conditions, respectively or (**C**,**D**) individual phenolic metabolites under pretreatment and cotreatment conditions. Cell proliferation was assessed by the MTT assay and is expressed as a percentage of untreated control cells (CTs). Mix Eq, equimolar mixture; Mix %C, mixture formulated according to the maximum brain concentrations of phenolic metabolites; Mix %T, mixture formulated according to the relative brain Tmax profile. Data are expressed as mean ± SD from 3 to 5 independent experiments. (*p* < 0.05; unpaired two-tailed Student’s *t*-test).

None of the three physiological mixtures, nor any of the compounds evaluated individually, exhibited cytotoxic effects at the concentrations tested (2.5, 5, and 10 μM) after 24 h of incubation, with cell viability consistently remaining above 90% relative to CT cells. As shown in Figure 1, treatment with the physiological mixtures alone at 10 μM did not significantly affect basal SH-SY5Y cell viability. Neither individual metabolites nor lower mixture concentrations affected cell viability. As expected, the treatment with H_2_O_2_ (200 μM) markedly decreased SH-SY5Y cell viability by around 60–30% compared with CT (Figure 1).

Pretreatment with Mix Eq, Mix %C, and Mix %T significantly improved cell viability following H_2_O_2_ exposure. This effect was statistically significant (*p* < 0.05) at 5 and 10 μM, whereas pretreatment at 2.5 μM provided only slight protection, although still significant (*p* < 0.05), for Mix %C and Mix %T (Figure 1A). Similarly, cotreatment with physiological mixtures attenuated the H_2_O_2_-induced cytotoxicity (*p* < 0.05) in a concentration-dependent manner (Figure 1B). Significant protection was observed for Mix Eq and Mix %C at 5 and 10 μM (*p* < 0.05), whereas lower concentrations produced only partial protection. Overall, pretreatment appeared to confer greater protection than cotreatment, suggesting that prior exposure to circulating phenolic metabolite mixtures enhances the resistance of SH-SY5Y cells to H_2_O_2_-induced oxidative stress. Among the 14 individual metabolites evaluated at 10 μM, hydroxytyrosol 4-*O*-sulfate (compound **1**), 2,4-dihydroxybenzoic acid (compound **8**), ellagic acid (compound **10**), dihydroresveratrol 3-*O*-glucuronide (compound **11**), 3-(2-hydroxyphenyl)propionic acid (compound **12**), resveratrol 3-*O*-sulfate (compound **13**), and 5-(3-hydroxyphenyl)valeric acid (compound **14**) significantly attenuated H_2_O_2_-induced cytotoxicity under pretreatment conditions (*p* < 0.05; Figure 1C). Under cotreatment conditions, hydroxytyrosol 4-*O*-sulfate (compound **1**), hydroxytyrosol 3-*O*-sulfate (compound **2**), 2,4-dihydroxybenzoic acid (compound **8**), 3-(3-hydroxyphenyl)propionic acid (compound **9**), 3-(2-hydroxyphenyl)propionic acid (compound **12**), and 5-(3-hydroxyphenyl)valeric acid (compound **14**) significantly improved cell viability compared with H_2_O_2_-treated cells (*p* < 0.05; Figure 1D). Nevertheless, none of the individual compounds reproduced the magnitude of protection previously observed for the physiological mixtures, particularly under pretreatment conditions. These results indicate that although certain metabolites may contribute more prominently to the overall neuroprotective response, the effects observed for the representative mixtures cannot be attributed exclusively to a single metabolite.

**Figure 2:**
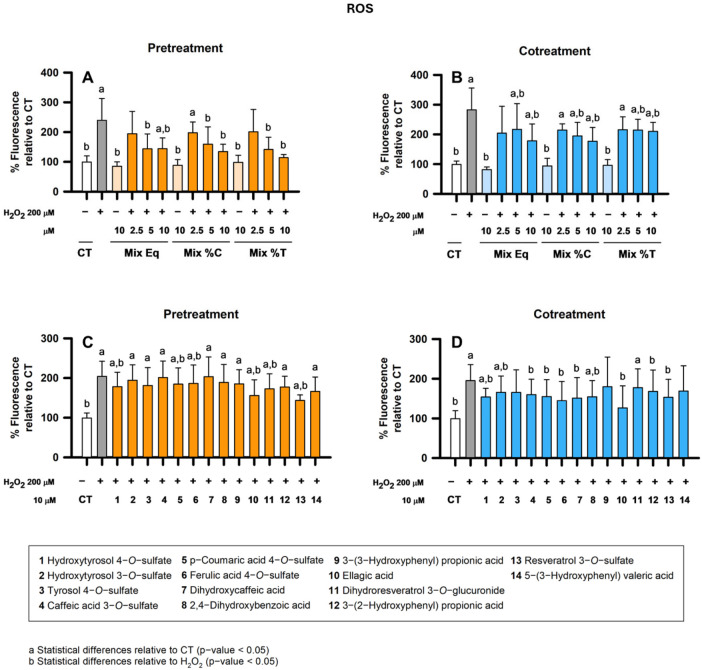
Effects of physiological phenolic metabolite mixtures and individual phenolic metabolites on H_2_O_2_-induced intracellular ROS production in SH-SY5Y cells. (**A**,**B**) Physiological mixtures under pretreatment and cotreatment conditions, respectively; (**C**,**D**) individual phenolic metabolites under pretreatment and cotreatment conditions, respectively. ROS levels were assessed by the H_2_DCFDA assay and are expressed relative to untreated control cells (CTs). Mix Eq, equimolar mixture; Mix %C, mixture based on maximum brain concentrations; Mix %T, mixture based on relative brain Tmax. Data are expressed as mean ± SD from 3 to 5 independent experiments. (*p* < 0.05; unpaired two-tailed Student’s *t*-test).

The effects of the three physiological mixtures and individual metabolites on H_2_O_2_-induced intracellular ROS levels are shown in Figure 2. Since H_2_O_2_ treatment reduced SH-SY5Y cell viability after 24 h of incubation, ROS data were normalized to MTT-derived viability values. In addition, treatment with the physiological mixtures or individual metabolites alone did not significantly modify basal ROS levels compared with CT cells.

As expected, H_2_O_2_ treatment remarkably boosted intracellular ROS levels in SH-SY5Y cells, yielding DCF-fluorescence intensity around 2.5–3-fold higher than those observed in CT cells (*p* < 0.05), whereas no effect was observed with the treatment with mixtures alone at the highest concentration (10 μM). Both pretreatment and cotreatment with Mix Eq, Mix %C, and Mix %T reduced intracellular ROS levels compared with H_2_O_2_-treated cells. Significant reductions were generally observed at 5 and 10 μM (*p* < 0.05), whereas 2.5 μM produced only modest effects. Overall, the antioxidant effect appeared more pronounced under pretreatment conditions, while most cotreatment groups still exhibited ROS levels significantly higher than those of CT cells (Figure 2A,B).

Among the individual metabolite treatments (10 μM), several compounds moderately reduced H_2_O_2_-induced intracellular ROS production in SH-SY5Y cells. Under pretreatment conditions, hydroxytyrosol 4-*O*-sulfate (compound **1**), *p*-coumaric acid 4-O-sulfate (compound **5**), ferulic acid 4-*O*-sulfate (compound **6**), ellagic acid (compound **10**), dihydroresveratrol 3-*O*-glucuronide (compound **11**), and resveratrol 3-*O*-sulfate (compound **13**) significantly decreased ROS levels compared with H_2_O_2_-treated cells (*p* < 0.05; Figure 2C). Similarly, under cotreatment conditions, hydroxytyrosol 4-*O*-sulfate (**1**), hydroxytyrosol 3-*O*-sulfate (**2**), caffeic acid 3-*O*-sulfate (**4**), *p*-coumaric acid 4-*O*-sulfate (**5**), ferulic acid 4-*O*-sulfate (**6**), dihydroxycaffeic acid (**7**), 2,4-dihydroxycaffeic acid (**8**), ellagic acid (**10**), 3-(2-hydroxyphenyl) propionic acid (**12**), and resveratrol 3-*O*-sulfate (**13**) significantly attenuated ROS production relative to the H_2_O_2_ group (*p* < 0.05; Figure 2D). Nevertheless, despite these reductions, intracellular ROS levels in most individual metabolite conditions remained significantly higher than those of CT cells, indicating only partial protection against oxidative stress. In contrast, the physiological mixtures consistently reduced ROS accumulation across the different experimental conditions evaluated.

### 2.2. Physiological Mixtures and Individual Circulating Phenolic Metabolites Exert Slight Anti-Inflammatory Effects on Inflamed Human HMC3 Microglial Cells

**Figure 3:**
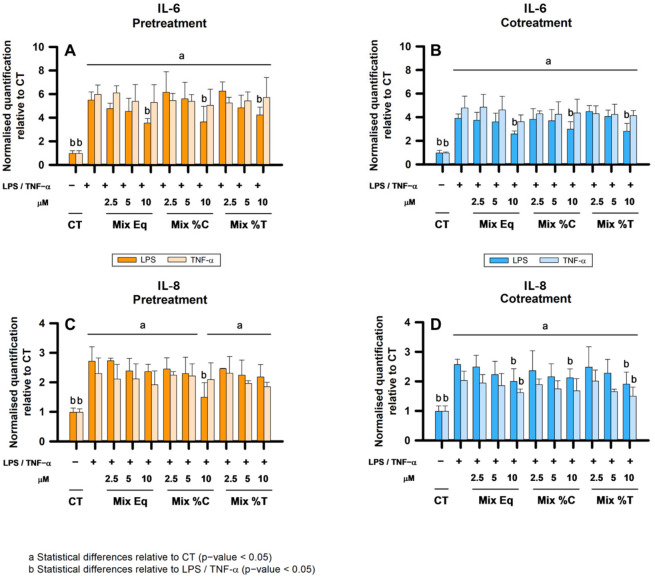
Effects of physiological phenolic metabolite mixtures on LPS- and TNF-α-induced IL-6 and IL-8 secretion in HMC3 cells. (**A**,**B**) IL-6 levels under pretreatment and cotreatment conditions, respectively; (**C**,**D**) IL-8 levels under pretreatment and cotreatment conditions, respectively. Cells were stimulated with LPS (500 ng/mL) or TNF-α (50 ng/mL) in the presence of the indicated mixtures (2.5, 5 and 10 µM). Cytokine levels are expressed relative to untreated control cells (CTs). Mix Eq, equimolar mixture; Mix %C, mixture based on maximum brain concentrations; Mix %T, mixture based on relative brain Tmax. Data are mean ± SD from 3 to 4 independent experiments. (*p* < 0.05; unpaired two-tailed Student’s *t*-test).

**Figure 4:**
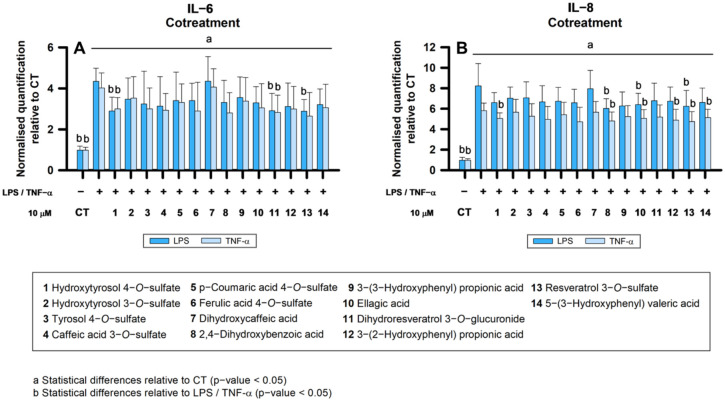
Effects of individual phenolic metabolites on LPS- and TNF-α-induced IL-6 and IL-8 secretion in HMC3 cells under cotreatment conditions. (**A**) IL-6 levels; (**B**) IL-8 levels. Cells were stimulated with LPS (500 ng/mL) or TNF-α (50 ng/mL) in the presence of the indicated individual phenolic metabolites (10 µM). Cytokine levels are expressed relative to CT cells. Data are mean ± SD from 3 to 4 independent experiments (*p* < 0.05; unpaired two-tailed Student’s *t*-test).

As expected, stimulation with LPS or TNF-α for 24 h markedly increased IL-6 and IL-8 release compared with CT cells (Figure 3 and Figure 4), confirming the induction of an inflammatory response in HMC3 microglia. The physiological mixtures produced only modest effects on cytokine production.

Both pretreatment and cotreatment with the three physiological mixtures (Mix Eq, Mix %C, and Mix %T) attenuated IL-6 production, being only statistically significant at 10 μM (*p* < 0.05) for LPS-stimulated HMC3 cells, but not for TNF-a (Figure 3A,B). Regarding IL-8 production under pretreatment conditions, only Mix %C at 10 μM significantly reduced cytokine levels at specific concentrations for LPS-stimulated HMC3 cells (*p* < 0.05; Figure 3C). Under cotreatment conditions, all three physiological mixtures significantly reduced IL-8 release in LPS-stimulated cells at 10 μM (*p* < 0.05), whereas Mix Eq and Mix %T also significantly decreased IL-8 production in TNF-α-stimulated cells (*p* < 0.05; Figure 3D).

Given the overall modest anti(neuro)-inflammatory effect observed for the mixtures, individual metabolites were subsequently evaluated under cotreatment conditions at 10 μM. Cotreatment was selected because it produced a greater number of significant effects on cytokine release than pretreatment, particularly for IL-8 production, thereby allowing a more comprehensive assessment of the potential contribution of individual metabolites to the anti-inflammatory activity observed for the physiological mixtures. Only a limited number of metabolites significantly modulated cytokine production in stimulated HMC3 cells, while cytokine levels generally remained significantly higher than those observed in CT cells (*p* < 0.05) (Figure 4). Hydroxytyrosol 4-*O*-sulfate (**1**) and dihydroresveratrol 3′-*O*-glucuronide (**11**) significantly reduced IL-6 release in both LPS- and TNF-α-stimulated cells (*p* < 0.05), whereas resveratrol 3-*O*-sulfate (**13**) significantly decreased IL-6 levels only in LPS-stimulated cells (Figure 4A). Regarding IL-8 production, 2,4-dihydroxybenzoic acid (**8**), ellagic acid (**10**), and resveratrol 3-*O*-sulfate (**13**) significantly reduced cytokine levels in both LPS- and TNF-α-stimulated cells (*p* < 0.05). In addition, hydroxytyrosol 4-*O*-sulfate (**1**), 3-(2-hydroxyphenyl)propionic acid (**12**), and 5-(3-hydroxyphenyl)valeric acid (**14**) significantly reduced IL-8 release in TNF-α-stimulated cells (*p* < 0.05; Figure 4B).

### 2.3. Physiological Phenolic Metabolite Mixtures Exert Modest Protective Effects Against TNF-α-Induced BBB Dysfunction

Following evaluation of the three physiological mixtures in microglial and neuroblastoma cells, their potential on the BBB was also assessed. Because endothelial cells are directly exposed to circulating metabolites, cells were treated under cotreatment conditions with the physiological mixtures and TNF-α.

First, the potential effects of the mixtures on BBB integrity under basal conditions were assessed by measuring transendothelial electrical resistance (TEER). None of the mixtures significantly altered TEER values compared with untreated control cells, indicating that they did not compromise endothelial monolayer integrity.

**Figure 5:**
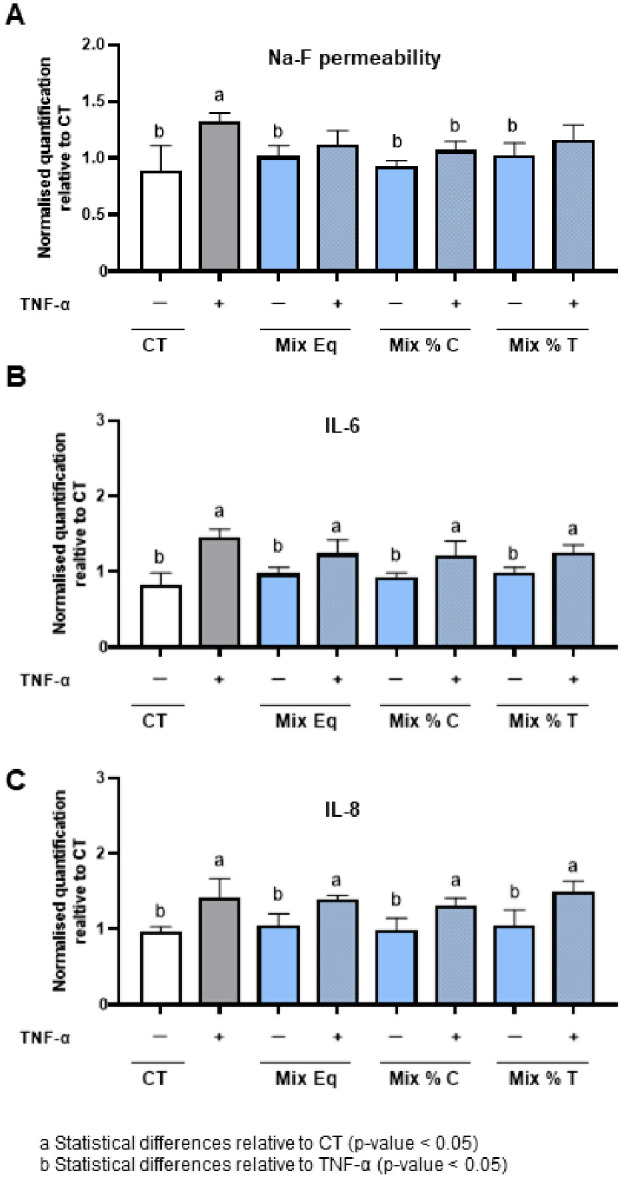
Effects of physiological phenolic metabolite mixtures on TNF-α-induced BBB dysfunction and inflammatory response in HBMECs. (**A**) Sodium fluorescein (Na-F) permeability; (**B**) IL-6 levels; and (**C**) IL-8 levels. Cells were cotreated with TNF-α (50 ng/mL) and the indicated physiological mixtures (10 µM). Na-F permeability and cytokine levels are expressed relative to untreated control cells (CTs). Mix Eq, equimolar mixture; Mix %C, mixture based on maximum brain concentrations; Mix %T, mixture based on relative brain Tmax. Data are mean ± SD from 3 independent experiments. (*p* < 0.05; unpaired two-tailed Student’s *t*-test).

BBB dysfunction was subsequently evaluated by measuring sodium fluorescein (Na-F) permeability. TNF-α significantly increased Na-F permeability compared with untreated control cells, confirming the disruption of endothelial barrier function. Cotreatment with the three physiological mixtures showed a modest tendency to attenuate the TNF-α-induced increase in Na-F permeability. However, only Mix %C significantly prevented this increase (Figure 5A).

To further characterize the effects of the physiological mixtures on the inflammatory response of BBB endothelial cells, IL-6 and IL-8 concentrations were measured in cell culture supernatants following TNF-α stimulation (Figure 5B,C). As expected, TNF-α significantly increased the release of both cytokines compared with CT cells, confirming the establishment of an inflammatory response in the endothelial monolayer. Under basal conditions, treatment with the physiological mixtures did not significantly affect IL-6 or IL-8 secretion. In TNF-α-stimulated cells, cotreatment with the mixtures did not significantly attenuate the increase in IL-6 release. Nevertheless, a modest numerical reduction in IL-6 concentrations was observed, particularly in cells cotreated with Mix %C, compared with TNF-α-stimulated cells. However, these differences did not reach statistical significance (Figure 5B). Similarly, none of the physiological mixtures significantly reduced TNF-α-induced IL-8 secretion. In contrast to the slight downward tendency observed for IL-6, no consistent reduction in IL-8 concentrations was detected across the different cotreatment conditions (Figure 5C).

## 3. Discussion

Neurodegenerative diseases (ND) are characterized by a multifactorial pathogenesis involving interconnected processes such as oxidative stress, chronic neuroinflammation, and blood–brain barrier (BBB) dysfunction [5,6,8]. Given the current lack of effective therapeutic interventions capable of preventing or slowing disease progression, increasing attention has been directed towards preventive approaches targeting early pathological mechanisms involved in neurodegeneration [6]. In this regard, dietary (poly)phenols and their circulating metabolites have attracted considerable interest because of their pleiotropic biological properties and their potential to modulate multiple processes implicated in ND, including oxidative stress, neuroinflammation, and alterations in BBB integrity [32,33].

Following dietary intake, most (poly)phenols undergo extensive phase I and phase II metabolism, together with gut microbiota-mediated biotransformation, resulting in a broad range of circulating phenolic metabolites rather than the parent compounds originally present in foods [34,35]. Some of these metabolites may reach systemic tissues, including the brain, at low but physiologically relevant concentrations and may collectively contribute to the biological effects associated with (poly)phenol-rich diets [27,31]. Therefore, assessing individual parent compounds at supraphysiological concentrations may provide limited information on the mechanisms underlying their potential health effects in vivo [19]. Instead, realistic combinations of circulating metabolites, with concentrations and relative proportions reflecting their physiological exposure, may offer a more biologically relevant approach.

Accordingly, here we evaluated the neuroprotective potential of three physiological phenolic metabolite mixtures, together with their individual constituent metabolites, designed according to previous reported plasma and brain metabolite profiles following a Mediterranean (poly)phenol-rich intervention [31].

Two exposure strategies were applied to determine whether the timing of metabolite availability could influence their neuroprotective activity. Under pretreatment conditions, SH-SY5Y cells were exposed to the mixture or individual metabolites for 6 h before the oxidative or inflammatory challenge, and treatments were maintained until 24 h later. This approach was intended to model a preventive scenario in which circulating metabolites are already present before the onset of cellular damage. In contrast, under cotreatment conditions, cells were simultaneously exposed to the test treatments and the insult for 24 h, modelling the potential ability of the metabolites to counteract oxidative or inflammatory damage as it occurs. The use of both approaches is relevant because dietary phenolic metabolites may be present either before or during exposure to endogenous or environmental oxidative [36].

In the present study, all three physiological mixtures protected SH-SY5Y cells against H_2_O_2_-induced oxidative damage, as evidenced by improved cell viability and reduced intracellular ROS accumulation. The protective effects were generally more evident at 5 and 10 µM, supporting a concentration-dependent response within the range tested. Pretreatment was generally associated with a greater improvement in cell viability, whereas cotreatment more consistently reduced intracellular ROS levels. This suggests that prior exposure to the mixtures may enhance cellular resilience to subsequent oxidative injury, while their simultaneous presence during H_2_O_2_ exposure may be particularly effective at limiting ROS accumulation and the early events associated with oxidative damage. These findings are partly consistent with a previous study in the same SH-SY5Y/H_2_O_2_ model insults. In that study, phenolic acids, gallic acid, and ellagic acid protected SH-SY5Y cells under both experimental approaches, whereas urolithins showed significant protection mainly under pretreatment conditions and did not exert protective effects during cotreatment [25]. Similarly, another in vitro study reported that microbial-derived phenolic acids identified after wine consumption, including 3,4-dihydroxyphenylacetic acid, hydroxyphenylpropionic acids, and hydroxyphenylacetic acid, protected SH-SY5Y cells against SIN-1-induced nitrosative stress at physiologically relevant concentrations ranging from 0.1 to 10 µM, supporting the ability of dietary-derived phenolic metabolites to influence intracellular pathways involved in neuronal stress and cell death [26]. Together, these observations indicate that the relative efficacy of preventive and concurrent exposure may depend on the metabolite profile, the biological endpoint evaluated, and the mechanisms underlying the response [37,38].

In the present study, although several individual metabolites exerted protective effects under specific conditions, none consistently reproduced the overall magnitude of protection or the reduction in intracellular ROS observed with the physiological mixtures. Consistent with our findings, an equimolar mixture of oleuropein, *p*-coumaric acid, and tyrosol protected neurons against H_2_O_2_- and paraquat-induced toxicity, reduced intracellular ROS and protein oxidation, whereas the individual compounds were ineffective or less effective within the same concentration range [39]. Moreover, in rats fed a high-fat diet, a mixture of d-limonene, gallic acid, ellagic acid, and *p*-coumaric acid produced the most favourable response in brain catalase activity and showed among the lowest brain levels of oxidation protein products (AOPPs), malondialdehyde (MDA), 4-hydroxynonenal (4-HNE), and total cyclooxygenase activity compared with the individual treatments [40]. This finding supports the relevance of evaluating circulating phenolic metabolites as physiologically occurring combinations, as metabolites with distinct chemical structures and cellular targets may exert complementary actions against oxidative stress-induced injury.

Oxidative stress and neuroinflammation are closely interconnected in the pathogenesis of ND. Persistent ROS accumulation can promote glial activation and the release of pro-inflammatory mediators, thereby establishing a loop of oxidative damage, inflammatory signalling, and neuronal injury [41,42]. Since microglia cells are central mediators of neuroinflammatory responses, the effects of the physiological mixtures were subsequently evaluated in HMC3 cells exposed to LPS or TNF-α.

In HMC3 cells, the anti-inflammatory effects of the physiological mixtures depended on the inflammatory stimulus, cytokine, concentration, and exposure strategy. All three mixtures showed a tendency to attenuate IL-6 release under both pretreatment and cotreatment conditions; however, a statistically significant reduction was only observed at 10 µM in LPS-stimulated cells. A similarly stimulus-dependent response was observed for IL-8. Under pretreatment conditions, only Mix %C at 10 µM significantly reduced IL-8 release in LPS-stimulated cells. In contrast, under cotreatment conditions, all three mixtures significantly decreased IL-8 production in LPS-stimulated cells at 10 µM, whereas Mix Eq and Mix %T also reduced IL-8 release in TNF-α-stimulated cells. Overall, these findings indicate that the mixtures exerted modest but significant anti-inflammatory effects in microglial cells, with the strongest responses generally observed at the highest concentration tested and under cotreatment conditions. The greater responsiveness of LPS-stimulated cells compared with TNF-α-stimulated cells suggests that the activity of the mixtures may depend on the inflammatory pathway activated by each stimulus. This interpretation is consistent with previous reports showing that diet-derived phenolic metabolites can display context-dependent anti-inflammatory activity depending on the inflammatory stimulus and cellular model employed. In particular, proposed protective effects of polyphenols against LPS-induced neuroinflammation are mainly related to modulation of the TLR4 pathway and microglial activation [30,43].

BBB impairment represents another relevant process linking neuroinflammation and neuronal damage. Increased BBB permeability can facilitate the entry of peripheral immune cells and circulating mediators into the brain parenchyma, thereby contributing to a pro-inflammatory environment and subsequent brain injury [44]. In addition, microglia can contribute to the BBB disruption by producing a variety of pro-inflammatory mediators, thereby enhancing BBB permeability and damage [45]. The effects of the physiological mixtures on BBB-related endpoints were evaluated under cotreatment conditions at 10 µM, as this strategy showed the most consistent responses in the previous cellular models [30,46]. TNF-α significantly increased sodium fluorescein (Na-F) permeability in HBMEC monolayers, confirming endothelial barrier impairment. Although all three mixtures showed a modest tendency to attenuate the TNF-α-induced increase in permeability, only Mix %C significantly prevented this effect. This finding suggests that the relative composition of the mixtures may influence their capacity to preserve endothelial barrier function under inflammatory conditions. TNFα reflects a physiologically relevant pro-inflammatory signal [47,48], although an increased IL-6 and IL-8 release in HBMECs after the insult, the cotreatment with the physiological mixtures did not significantly attenuate cytokine secretion. A modest reduction in IL-6 levels was observed, particularly in cells cotreated with Mix %C, whereas no consistent reduction in IL-8 release was detected. The lack of significant cytokine modulation contrasts with the protective effect of Mix %C on Na-F permeability, suggesting that its beneficial action on endothelial barrier integrity may not depend primarily on regulation of IL-6 and IL-8 secretion. This interpretation is consistent with previous evidence indicating that TNF-α can compromise brain endothelial barrier function through mechanisms involving tight junction proteins, cytoskeletal remodelling, and inflammatory signalling pathways, including NF-κB and MAPK activation. For instance, in an in vitro study using brain microvascular endothelial cells, TNF-α increased IL-6 and IL-8 release and selected polyphenols differentially modulated cytokine production and endothelial activation markers. Notably, some compounds produced only non-significant reductions in cytokine release, highlighting the compound- and endpoint-dependent nature of endothelial responses to polyphenols [49]. Therefore, the effect of Mix %C on Na-F permeability may involve mechanisms related to the preservation of endothelial junctional integrity or cytoskeletal regulation rather than a broad suppression of cytokine secretion.

Among the three formulations, Mix %C was the only mixture that significantly attenuated TNF-α-induced BBB hyperpermeability and also showed favourable effects in several oxidative stress and inflammatory endpoints. Remarkably, this mixture was characterized by high proportions of hydroxytyrosol sulfates, *p*-coumaric acid sulfate, 2,4-dihydroxybenzoic acid and 5-(3-hydroxyphenyl) valeric acid. Hydroxytyrosol and its derived metabolites have been associated with antioxidant and anti-inflammatory effects in experimental and human studies. In particular, hydroxytyrosol administration has been reported to regulate cellular components involved in neuroinflammatory responses and metabolic homeostasis in a model of hyperglycemia-induced cortical damage [50]. In addition, hydroxytyrosol supplementation improved antioxidant and inflammatory status in individuals with overweight and prediabetes, with these effects being associated with increased urinary levels of hydroxytyrosol sulfate metabolites [51,52].

Similarly, 2,4-dihydroxybenzoic acid, also known as resorcylic acid, may be of interest because it improved survival and neuropathological alterations in a mouse model of CoQ deficiency-associated encephalopathy by reducing astrogliosis and neuroinflammation-related gene expression, suggesting that this metabolite may contribute through metabolic and anti-inflammatory mechanisms beyond direct antioxidant activity [53]. Nevertheless, the biological effects of a physiological mixture cannot be directly attributed to any individual constituent, and further studies will be required to identify the metabolites or even interactions responsible for the observed effects.

Several limitations should be acknowledged. First, this study was conducted using in vitro cellular models; therefore, the observed effects require validation in more complex preclinical models and, ultimately, in human studies. Second, although the mixtures were designed according to previously reported brain metabolite profiles, circulating phenolic metabolites do not necessarily coexist at identical concentrations or time points in vivo anytime. Thus, the selected formulations represent a physiologically informed approximation rather than a complete reproduction of postprandial metabolite dynamics. Third, the molecular mechanisms underlying the protective effects were not directly investigated. Future studies should assess antioxidant defence systems, mitochondrial function, apoptosis-related pathways, microglial signalling, tight junction proteins, and endothelial cytoskeletal regulation to better define the mechanisms involved in the effects of the mixtures. Fourth, the cellular uptake and metabolic fate of the administered metabolites were not evaluated. Several studies have reported that HBMEC, SH-SY5Y, and HMC3 cells possess the capacity to metabolize phenolic compounds or other xenobiotics. Therefore, it cannot be excluded that the evaluated compounds underwent cellular uptake, deconjugation, conjugation, or further biotransformation, and that the resulting compounds contributed to the observed biological effects. Moreover, structural differences among metabolites, particularly their conjugation as sulfates or glucuronides, may influence their cellular uptake, thereby contributing to differences in their biological activity [54]. However, characterizing these processes was beyond the scope of the present study and should be addressed in future investigations.

## 4. Materials and Methods

### 4.1. Materials and Reagents

Trypan blue, 3-(4,5-dimethyl-2-thiazolyl)-2,5-diphenyl-2H-tetrazolium bromide (MTT), hydrogen peroxide solution, phosphate-buffered saline (PBS), dimethylsulfoxide (DMSO), 2,4-dihydroxybenzoic acid, and ellagic acid were purchased from Sigma-Aldrich (St. Louis, MO, USA). Dihydroresveratrol 3-*O*-glucuronide and resveratrol 3-*O*-sulfate were obtained as previously described [55]. Hydroxytyrosol 4-*O*-sulfate, hydroxytyrosol 3-*O*-sulfate, tyrosol 4-*O*-sulfate, *p*-coumaric acid 4-*O*-sulfate, 3-(3-hydroxyphenyl) propionic acid and 5-(3-hydroxyphenyl)-valeric acid were purchased from Toronto Research Chemicals (Toronto, ON, Canada). Ferulic acid 4-*O*-sulfate was provided by Biosynth (Compton, Berkshire, UK), whereas 3-(2-hydroxyphenyl) propionic acid was obtained from Fluorochem (Hadfield, Derbyshire, UK). Dihydrocaffeic acid and caffeic acid 3-*O*-sulfate were kindly provided by Dr. Nunes Dos Santos (NIMSB, Lisbon, Portugal). Ultrapure water was obtained from a Milli-Q purification system (Millipore Corp., Bedford, MA, USA) and used throughout the study.

### 4.2. Cell Culture

The human microglia cell line (HMC3) was kindly provided by Dr Nunes Dos Santos (NOVA Medical School, Lisbon, Portugal) and was cultured as recommended by the American Type Culture Collection (ATCC) in Dulbecco’s modified Eagle’s medium (DMEM) enriched with 10% *v*/*v* fetal bovine serum (FBS), and supplemented with 2 mM glutamine and 1% *v*/*v* antibiotic solution (100 U mL^−1^ penicillin and 100 μg mL^−1^ streptomycin) (Gibco, Invitrogen S.A., Barcelona, Spain). The human neuroblastoma cell line SH-SY5Y was purchased from the European Collection of Authenticated Cell Cultures (ECACC) (Salisbury, UK). Cells were cultured as recommended by the ECACC in Ham’s F12:EMEM (EBSS) (1:1) with 15% FBS, and supplemented with 2 mM L-glutamine, 1% *v*/*v* non-essential amino acids, and 1% *v*/*v* antibiotic solution. The human brain microvascular endothelial cells (HBMECs), kindly provided by Prof. Kwang Kim (John Hopkins University, Baltimore, MD, USA) in 2008, was used as a simplified and validated in vitro model of the BBB, and were grown in RPMI medium (Sigma) with the addition of 10% FBS and 10% Nu-Serum IV (BD Biosciences, Madrid, Spain), and supplemented with 2 mM L-glutamine, 1% *v*/*v* non-essential amino acids, 1 mM sodium pyruvate and 1% *v*/*v* antibiotic solution (Gibco, Invitrogen S.A., Barcelona, Spain). All cell lines were incubated at 37 °C in a humidified atmosphere of 95% air/5% CO_2_.

### 4.3. Preparation of Physiological Phenolics Metabolites Mixtures and Cell Treatments

The phenolic metabolite mixtures used in this study were designed based on previously reported in vivo data describing the metabolic profile and brain distribution of circulating phenolic compounds after the intake of a Mediterranean (poly)phenol-rich mixture in Sprague-Dawley rats [31]. Specifically, the selection of metabolites and their relative proportions were established according to the compounds identified and quantified in perfused brain tissues. Of the 19 metabolites detected in the animal study, the 14 included in the present study were those for which authentic standards were available, allowing their reliable quantification, and that were available in sufficient amounts to perform both the mixture and individual compound experiments. A detailed list of the metabolites included in each mixture, together with their corresponding concentrations, is provided in Table 1.

Briefly, three physiologically relevant mixtures were prepared: (i) Mix Eq, containing all selected metabolites at equal concentrations, resulting in final total concentrations of 2.5, 5, or 10 μM; (ii) Mix %C, formulated to reproduce the relative proportions of each metabolite as observed in vivo, based on their maximum concentrations (Cmax) detected in brain tissue; and (iii) Mix %T, formulated according to the relative abundance of metabolites associated with their respective time-to-maximum concentration (Tmax) profiles in vivo, with the aim of reflecting a more dynamic physiological exposure scenario. Individual metabolites were also evaluated at concentrations of 2.5, 5, and 10 μM.

Both physiological mixtures and individual compounds were solubilized in DMSO and were filter-sterilized (0.2 μm) prior to their addition to the medium cells. The final concentration of DMSO in the culture medium did not exceed 0.5% (*v*/*v*). Vehicle control cells (CTs) containing the corresponding concentration of DMSO were included in all experiments.

### 4.4. Cell Viability in Oxidative Stress-Induced SH-SY5Y Cells

The induction of oxidative stress in SH-SY5Y cells was carried out following a protocol previously described by González-Sarrías et al. (2017) [25], with slight modifications. Briefly, SH-SY5Y cells were seeded in 96-well plates at a density of 1.5 × 10^4^ cells per well and incubated for 24 h to allow cell attachment and stabilization. To establish the oxidative stress model, cells were exposed to increasing concentrations of H_2_O_2_ (20–400 μM) for 24 h. A concentration of 200 μM was selected for subsequent experiments, as it resulted in approximately 40–60% reduction in cell viability compared with CT cells.

**Figure 6:**
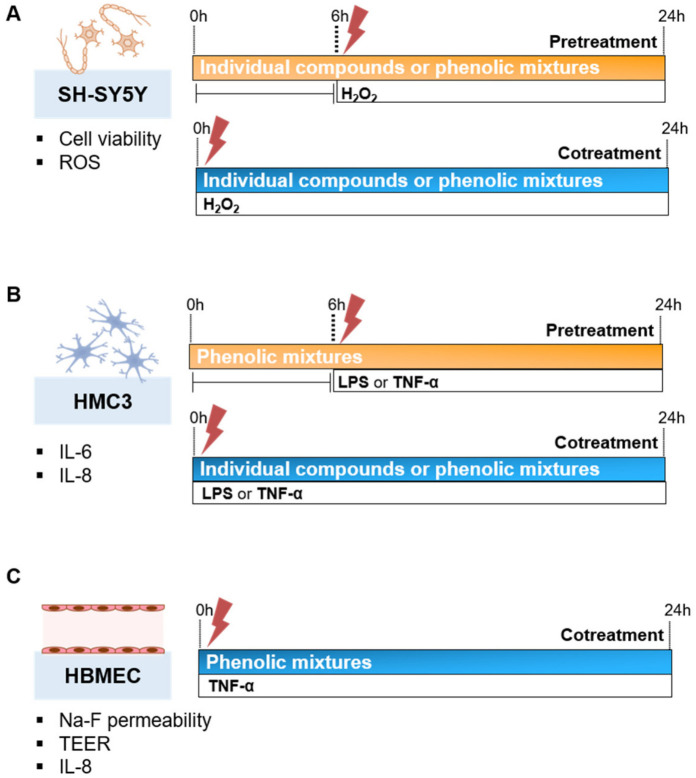
Schematic representation of the treatments applied to the different cellular models. (**A**) SH-SY5Y cells were exposed to individual phenolic metabolites or physiological mixtures either before hydrogen peroxide (H_2_O_2_) challenge (pretreatment, orange) or simultaneously with H_2_O_2_ (cotreatment, blue). (**B**) HMC3 cells were treated with the physiological mixtures before stimulation (pretreatment) or with individual metabolites or mixtures simultaneously (cotreatment) with lipopolysaccharide (LPS) or tumour necrosis factor-alpha (TNF-α). (**C**) HBMEC monolayers were cotreated with the physiological mixtures and TNF-α.

To evaluate the neuroprotective effects of the physiological metabolite mixtures and individual phenolic metabolites, two treatment strategies were applied. In the pretreatment condition, cells were incubated with the test treatments (2.5, 5, or 10 μM) for 6 h prior to oxidative challenge. Subsequently, H_2_O_2_ (200 μM) was added to the culture medium without removing the treatments, and cells were further incubated for 24 h. In the cotreatment condition, cells were simultaneously exposed to both the test treatments (2.5, 5, or 10 μM) and H_2_O_2_ (200 μM) for 24 h without prior incubation (Figure 6A).

Cell viability following each treatment was determined using the MTT reduction assay, as previously reported by González-Sarrías et al. (2022) [56]. The cytotoxic effect was expressed as the percentage of viable cells relative to CT (0.5% DMSO, set at 100%). Data are presented as the mean ± standard deviation (SD) from at least five independent experiments.

### 4.5. Measurement of Reactive Oxygen Species (ROS) in SH-SY5Y Cells

ROS levels were determined following the method previously described by González-Sarrías et al. (2017) [25], using the ROS-sensitive probe 2′,7′-dichlorodihydrofluorescein diacetate (H2DCFDA; Sigma, St. Louis, MO, USA). After treatments on H_2_O_2_-induced SH-SY5Y cells (Figure 6A), the medium was removed and incubated with 25 μM H2DCFDA for 2 h at 37 °C in the dark. Once inside the cells, H2DCFDA is oxidized by ROS to generate the highly fluorescent molecule 2′,7′-dichlorofluorescein (DCF). Fluorescence intensity was measured at an excitation wavelength of 495 nm and an emission wavelength of 520 nm using a multimode microplate reader (FLUOstar Omega, BMG Labtech, Ortenberg, Germany).

### 4.6. Cytokine Analysis in HMC3 Microglia Cells

Cytokine production was assessed following a previously described methodology [30]. Briefly, HMC3 cells were plated in 24-well plates at a density of 7.5 × 10^4^ cells per well and allowed to grow for 48 h. Once sub-confluent, cells were exposed for 24 h to TNF-α (50 ng/mL) or LPS (500 ng/mL) in the presence or absence of the physiological metabolite mixtures or individual phenolic metabolites (2.5, 5, or 10 μM) (Figure 6B). Cells treated with 0.5% DMSO alone (CT) were included as negative controls.

Following treatment, culture supernatants were collected and stored at −80 °C until further analysis. The concentrations of IL-6 and IL-8 were determined using commercially available ELISA kits (PeproTech, Rocky Hill, NJ, USA), with sensitivity limits of 24 pg/mL and 16 pg/mL, respectively. Absorbance readings were obtained using a microplate reader (Infinite M200, Tecan, Grödig, Austria), and cytokine levels were calculated based on standard calibration curves.

To exclude potential cytotoxic effects of the treatments, cell viability was assessed after 24 h using the MTT assay as described above. Results are expressed as mean ± SD from at least three independent experiments (n = 3).

### 4.7. Inflammation-Induced Disruption of the BBB In Vitro

An in vitro BBB model was used using HBMECs, following a previously established protocol [30,57]. Briefly, the HBMECs were cultured in 12-well Transwell^®^ inserts (12 mm, 0.4 µm pore polyester membrane; Corning, Madrid, Spain) for 7 days at 37 °C in a humidified incubator with 5% CO_2_ to allow the formation of confluent monolayers mimicking the BBB.

To induce endothelial inflammation and BBB dysfunction, HBMECs were exposed to 50 ng/mL of TNF-α in the apical compartment for 24 h and co-incubated with the physiological metabolite mixtures (Mix Eq, Mix %C or Mix %T at 10 μM (Figure 6C). CT cells (0.5% DMSO) were used as negative controls, whereas cells exposed to TNF-α alone (50 ng/mL) served as positive controls for endothelial inflammation and BBB dysfunction.

Following treatment, culture supernatants were collected and stored at −80 °C until analysis. The concentrations of IL-6 and IL-8 were quantified using commercially available ELISA kits (PeproTech, Rocky Hill, NJ, USA) according to the manufacturer’s instructions.

BBB integrity of the cell monolayers was confirmed by monitoring transendothelial electrical resistance (TEER) at the start (0 h) and end (24 h) of the experiment using an EVOM2 Epithelial Volt Ohm Metre (World Precision Instruments, Inc., Sarasota, FL, USA). In addition, the paracellular permeability of the endothelial monolayer was evaluated using Na-F (molecular weight: 376 Da) as a low-molecular-weight permeability marker [30,58]. Following treatment, monolayers were washed with HBSS and incubated with Na-F (10 μg/mL). Inserts were transferred to fresh HBSS-containing wells every 20 min for a total period of 1 h. Samples collected from the basolateral compartment were analyzed using a CLARIOstar microplate reader (BMG LABTECH, Ortenberg, Germany), and the endothelial permeability coefficient (Pe, cm/min) was measured and calculated as previously described [30,55,59]. Results were expressed relative to CT control cells.

### 4.8. Statistical Analysis

Data are presented as mean ± SD from three to five independent experiments. Statistical analyses were performed using GraphPad Prism version 10.1.1 (GraphPad Software, San Diego, CA, USA). For each cell model and experimental condition, planned comparisons were performed between each treatment and the corresponding negative control cells (vehicle-treated cells, 0.5% DMSO), and between each treatment and the corresponding positive control cells exposed to H_2_O_2_, LPS, or TNF-α, as appropriate. Data distribution was assessed for normality using the Shapiro–Wilk test. Comparisons were analyzed using unpaired two-tailed Student’s *t*-tests. Statistical significance was set at *p* < 0.05. In the figures, the letter “a” indicates significant differences compared with the negative control, whereas the letter “b” indicates significant differences compared with the corresponding positive stimulus control.

## 5. Conclusions

The present study shows that physiological mixtures of circulating phenolic metabolites can protect human cellular models from oxidative and inflammatory insults relevant to neurodegeneration. In SH-SY5Y cells, all three mixtures improved cell viability and reduced H_2_O_2_-induced ROS accumulation, with effects that were generally more consistent and broader than those observed for the individual metabolites. In HMC3 microglial cells, the mixtures exerted moderate anti-inflammatory effects that depended on the inflammatory stimulus, cytokine, concentration, and treatment strategy, with the strongest effects observed under cotreatment conditions, particularly in LPS-stimulated cells. In addition, some conditions significantly attenuated TNF-α-induced Na-F permeability in an in vitro BBB model.

Overall, these findings support the relevance of studying phenolic metabolites as physiologically occurring combinations rather than as isolated compounds. The greater activity observed for the mixtures suggests that metabolites with different chemical structures and biological targets may act in a complementary manner to limit oxidative stress, microglial inflammatory responses, and endothelial barrier impairment. Although further mechanistic and in vivo studies are needed, these results provide evidence that circulating phenolic metabolite mixtures may contribute to the neuroprotective effects associated with Mediterranean (poly)phenol-rich dietary patterns.

## Figures and Tables

**Table 1:** Physiological mixtures of circulating phenolic compounds detected in the perfused brain after the intake of a Mediterranean (poly)phenol-rich mixture [31], using equal concentrations (Mix Eq), percentages according to the Cmax values of each one (Mix %C) or percentages according to the Tmax (Mix %T).

	Compound	Mix Eq	Mix %C	Mix %T
**1**	Hydroxytyrosol 4-*O*-sulfate	7.14%	21.91%	3.47%
**2**	Hydroxytyrosol 3-*O*-sulfate	7.14%	15.52%	0.96%
**3**	Tyrosol 4-*O*-sulfate	7.14%	0.46%	2.05%
**4**	Caffeic acid 3-*O*-sulfate	7.14%	4.56%	1.20%
**5**	*p*-Coumaric acid 4-*O*-sulfate	7.14%	12.78%	0.33%
**6**	Ferulic Acid 4-*O*-sulfate	7.14%	0.91%	20.74%
**7**	Dihydrocaffeic acid	7.14%	3.19%	28.88%
**8**	2,4-Dihydroxybenzoic acid	7.14%	19.63%	1.58%
**9**	3-(3-Hydroxyphenyl) propionic acid	7.14%	4.56%	3.28%
**10**	Ellagic acid	7.14%	0.50%	0.41%
**11**	Dihydroresveratrol 3-*O*-glucuronide	7.14%	1.83%	3.74%
**12**	3-(2-Hydroxyphenyl) propionic acid	7.14%	2.74%	21.44%
**13**	Resveratrol 3-*O*-sulfate	7.14%	0.46%	0.00%
**14**	5-(3-Hydroxyphenyl) valeric acid	7.14%	10.95%	11.91%

## Data Availability

The original contributions presented in this study are included in the article. Further inquiries can be directed to the corresponding author.

## Abbreviations

The following abbreviations are used in this manuscript:

BBB	Blood–brain barrier
DCF	2′,7′-dichlorofluorescein
DMSO	Dimethyl sulfoxide
H2DCFDA	2′,7′-dichlorodihydrofluorescein diacetate
IL-6	Interleukin 6
IL-8	Interleukin 8
LPS	Lipopolysaccharide
Mix Eq	Mix of metabolites at equal concentrations
Mix %C	Mix of metabolites based on their maximum concentrations
Mix %T	Mix of metabolites based on their time-to-maximum concentration
Na-F	Sodium fluorescein
ND	Neurodegenerative disease
ROS	Reactive oxygen species
SD	Standard deviation
TEER	Transendothelial electrical resistance
TNF-α	Tumour necrosis factor alpha
